# The Effect of Dexmedetomidine on Cognitive Function and Protein Expression of A*β*, p-Tau, and PSD95 after Extracorporeal Circulation Operation in Aged Rats

**DOI:** 10.1155/2018/4014021

**Published:** 2018-01-16

**Authors:** Ying Zhang, Yong Lin, Qing Liu, Xuemei Yuan, Anqiong Mao, Yuexin Liu, Qun Li, Jie Zheng, Bin Hu, Fengxu Yu

**Affiliations:** ^1^Department of Anesthesiology, Affiliated Traditional Chinese Medicine Hospital of Southwest Medical University, Luzhou, Sichuan Province, China; ^2^Department of Emergency, Affiliated Traditional Chinese Medicine Hospital of Southwest Medical University, Luzhou, Sichuan Province, China; ^3^Department of Cardiovascular Surgery, Affiliated Hospital of Southwest Medical University, Luzhou, Sichuan Province, China

## Abstract

Postoperative cognitive dysfunction (POCD) is a kind of serious neurologic complications and dexmedetomidine has a certain effect on POCD. However, functional mechanism of dexmedetomidine on POCD still remains unclear, so the research mainly studied the effect of dexmedetomidine on cognitive function and protein expression in hippocampus and prefrontal cortex cerebrospinal fluid after extracorporeal circulation operation in aged rats. We Found that, compared with POCD group, the cognitive function was improved in POCD + Dex group. We speculate that dexmedetomidine could improve the cognitive function after extracorporeal circulation operation in aged rats and A*β*, p-Tau, and PSD95 protein might have contributed to this favorable outcome.

## 1. Introduction

Postoperative cognitive dysfunction (POCD) is a kind of neurologic complications, characterized by impaired memory, learning difficulty, and focus ability for several weeks or even longer clinically [1]. POCD is associated with early exit from the work force, decreased quality of life, and premature mortality [1, 2]. It is reported that approximately 6% of adult patients developed POCD within 3 months after surgery, while elderly patients with POCD can reach 13% [2]. In the United States, elderly patients account for more than 40% of the total surgery. Hence, with the arrival of an aging society, POCD will become a main concern after surgery [3]. However, there were only few hypotheses on the mechanism of POCD, including inflammation, central cholinergic system dysfunction, and neuronal apoptosis. Therefore, it is very necessary and meaningful to further explore the mechanism of POCD to find therapeutic clues for the treatment of POCD.

Dexmedetomidine is a highly selective alpha-2 adrenergic receptor agonist, with the efficacy of sedation, analgesia, and sympathetic blocking [4]. It can also reduce the stress response and activate cholinergic anti-inflammatory pathway [5]. Moreover, it can also reduce catecholamine levels, improve cerebral oxygen supply and blood perfusion in ischemic area, and play a critical role in neural protection [6, 7]. Recently, some studies also have supported that when POCD occurred, treatment with dexmedetomidine could relieve the behavior of POCD through protecting neuronal function [8, 9]. However, the functional mechanism of this favorable outcome still remains unclear.

Therefore, in this study, we established the animal model of POCD in aged rats through extracorporeal circulation surgery, to investigate the effect and possible mechanism of dexmedetomidine on cognitive function.

## 2. Materials and Methods

### 2.1. Animals, Grouping, and Model Establishment

All of the animal experiments were approved by the Animal Care and Use Committee of Southwest Medical University and were performed in according to the guidelines established by the Chinese Association for Laboratory Animal Sciences. 90 Sprague-Dawley aged rats, aged 18~20 months, weight 400~550 g, were purchased from and kept in Animal Centre of Southwest Medical University. All the animals were trained on the Morris water-maze test to exclude the abnormal behavior rats for one week before the experiment. Finally, 6 rats were excluded and 84 rats were selected.

The 84 rats were randomly divided into 3 groups, including control group, POCD group, and POCD + Dex group, with 28 rats in each group. The extracorporeal circulation operation after intraperitoneal injection of 2 ml 0.9% normal saline was performed on the rats from POCD group. The extracorporeal circulation operation after intraperitoneal injection of 50 ug/kg dexmedetomidine dissolved in 2 ml and 0.9% normal saline was performed on the rats from POCD + Dex group. Any intervention was not accepted in the rats from control group.

The CPB model was conducted according to the method reported previously [10, 11]. Animals in POCD group and POCD + Dex group were anesthetized with chloral hydrate and isoflurane in 60% oxygen, and the anesthesia was maintained with 2.0% to 2.5% isoflurane and intravenous fentanyl (10 ug/kg). After orotracheal intubation with a 14 G cannula, the animals were mechanically ventilated with a tidal volume of 10 mL/kg and respiratory rate of 60 to 65 beats/min. Animals were additionally heparinised (250 IU/kg) after intubation in the right jugular vein and the tail artery. The tail artery was cannulated for blood pressure monitoring and blood sampling. The extracorporeal circulation circuit was composed of venous catheters, a peristaltic pump, a rat oxygenator, and polyethylene tubing (1.6 mm inner diameter) (Xijing Medical Supplies Co., Ltd.). The circle was initiated with 4 ml prefilling liquid used for precharging polyethylene tubing (2.2 ml ringer lactate solution, 1.4 ml hydroxyethyl starch, 0.2 ml 20% mannitol, and 0.2 ml 5% sodium bicarbonate). At the end of extracorporeal circulation, protamine (1.5 mg/kg intravenously) was administered, cannulae were removed, and wounds were sutured. A spontaneous breathing is the major diagnostic test to determine whether rats can be successfully extubated.

### 2.2. Morris Water-Maze Test

Before and after extracorporeal circulation operation, the rats from three groups were examined by the Morris water-maze test. In the whole experimental period, the water tank, mobile platform, perimeter marker position, and experimental time were fixed. The stainless steel pool was divided into four quadrants, and the movable platform located in the first quadrant. Five days for a training cycle, each rat was trained 4 times each day to record the average daily escape latency. In the first day, the rats were placed on the platform for 30 seconds and all the rats were adapted to the environment after the completion of the formal experiment, respectively. If the rat did not reach the platform for 120 seconds, it would be guided onto the platform and stayed on the platform for 10 seconds. The training record of the rats was 120 seconds.

After training experiment, the platform was removed, the opposite quadrant with the angle of 180 degrees from the quadrant of the original platform was as the point of entry, and the frequency of crossing the original platform was recorded in 120 seconds.

### 2.3. Shuttle Box Test

After extracorporeal circulation operation, passive avoidance memory test of the rats from three groups was examined by the shuttle box test. There were two illuminated and dark compartments in the shuttle box apparatus (27 × 14.5 × 14 cm). The device included a copper fence with electricity in the bottom of the box as unconditioned stimulus, a noise generator, and light source as conditioned stimulus on the top of the box. Each rat was placed in the illuminated chamber for 10 min without the electric shock to eliminate exploratory reflection. Then, rats were placed individually in the illuminated compartment with noise stimulation for 5 s and the entering delay of each rat in the dark chamber was recorded as initiative avoiding latency. After ten min, an electrical single shock (50 Hz, 0.2 mA, 3 s) was delivered through a copper fence without noise and light stimulation. The delay of fleeing to safety was recorded as passive avoiding latency. The times of avoiding with unconditioned and conditioned stimulus both were recorded. The first six days were the training time, and the results of avoiding latency at the seventh day were recorded.

### 2.4. Open Field Test

After extracorporeal circulation surgery, the rats from three groups were examined by the open field test. All the rats adapted to the environment for half an hour before the experiment, then the rat was seized and lifted gently in the open field test box.

The light source was fixed at the side of the box, the experiment time was selected as 5 min, and the activities of the rats were recorded. While the forelegs were off of the ground, which was recorded as 1 time positive activity. In order to avoid the residual odor of the previous rat, the feces and urine of rats were cleaned at the end of each experiment with 75% alcohol.

### 2.5. Cerebrospinal Fluid Collection and the Expression of A*β* Protein, PSD95, and p-Tau Protein

Cerebrospinal fluid was collected with standard methods [12]. In brief, the dorsal neck skin of anesthetized rat was disinfected. After exposure the membrane of atlantooccipitalis posterior, the cerebrospinal fluid was collected slowly through foramen magnum via needle connected to a draw syringe.

The concentrations of A*β* protein, PSD95, and p-Tau protein in cerebrospinal fluid were determined by enzyme-linked immunosorbent assay. The A*β* protein, PSD95, and p-Tau protein antibodies (Wako Pure Chemical Industries, Ltd., Japan) were added to the cerebrospinal fluid to form an immune complex, which then reacted with the substrates. The color of the reaction was positively correlated with the concentration of A*β* protein, PSD95, and p-Tau protein. The concentration of A*β* protein, PSD95, and p-Tau protein was calculated, according to the sample absorbance generated from plate reader (Infinite® 200 Pro NanoQuant, Tecan, Switzerland).

### 2.6. Brain Tissue Collection and the Immunohistochemistry Method for Detecting the Expression of A*β* Protein, PSD95, and p-Tau Protein

The deeply anesthetized rats were perfused with 0.9% NaCl and 4% paraformaldehyde, respectively. The fixed brain tissue was dehydrated and the paraffin section (5 *μ*m) was prepared. The A*β*, PSD95, and p-Tau protein positive neurons in target brain region were visualized by immunohistochemistry conducted according to instructions (Wako Pure Chemical Industries, Ltd., Japan). The positive cells showed brown cytoplasm where the antigens were located. The number of positive cells was calculated from five different fields of each slide under the microscope.

### 2.7. Statistical Analysis

The experimental data were processed by SPSS 19 statistical software. Data were described as mean ± standard deviation. Experiment parameters between the 2 groups were compared with the independent sample *t*-test. Experiment parameters among 3 groups were compared by analysis of ANOVA; multiple samples repeated measurement data were analyzed by repeated measures analysis of ANOVA; comparison among groups was compared by LSD (Least significant difference) test; count data between groups were compared by chi square test. *P* < 0.05 was considered as statistical significance.

## 3. Results

### 3.1. The Effects of Dexmedetomidine on Cognitive Dysfunction in Aged Rats From the 31st to 35th Days after Operation

**Figure 1:**
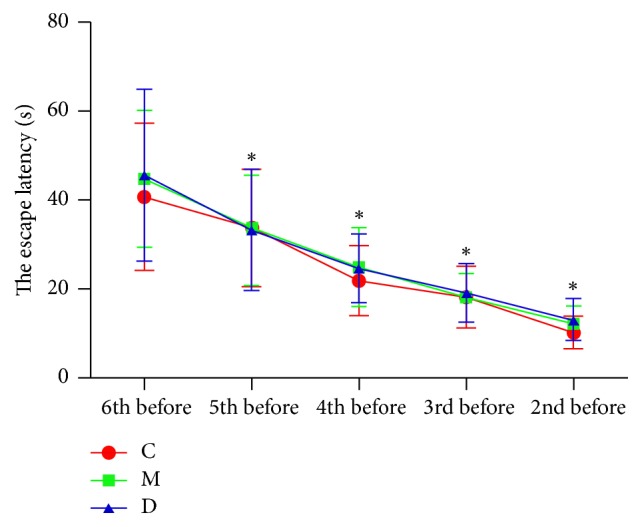
The comparison of the escape latency of rats in three groups before operation. C: control group; M: POCD group; D: POCD + Dex group. ^*∗*^Compared with 6th day before operation, *P* value < 0.05.

**Figure 2:**
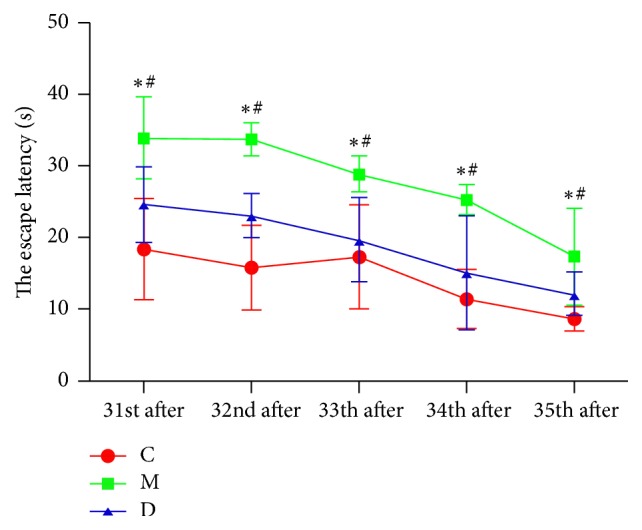
The comparison of the escape latency of rats in three groups after operation C: control group; M: POCD group; D: POCD + Dex group; ^*∗*^compared with control group, ^#^compared with POCD group. *P* < 0.05 was considered as statistical significance.

Before operation, in Morris water-maze test, the escape latency for 5 continuous days decreased with extended learning time (*P* < 0.05). However, the difference in escape latency among groups had no statistical significance (*P* > 0.05), as shown in Figure 1. Compared with control group, the escape latency increased in POCD group from the 31st to 35th days after operation (*P* < 0.05). In addition, compared with POCD group, the escape latency decreased in POCD + Dex group from the 31st to 35th days after operation (*P* < 0.05), as shown in Figure 2. Finally, after operation, in Morris water-maze test, compared with control group, the frequency of crossing the original platform decreased in POCD group (*P* < 0.05). And, compared with POCD group, the frequency of crossing the original platform increased in POCD + Dex group (1.2 ± 0.9 versus 4.8 ± 1.3 versus 3.2 ± 1.4, *F* = 109.11, *P* < 0.05).

After operation, in shuttle box test, compared with control group, the time of avoiding decreased and latency of the initiative and passive avoiding increased in POCD group (*P* < 0.05), as shown in Table 1. However, compared with POCD group, the time of avoiding increased, and latency of the initiative and passive avoiding decreased in POCD + Dex group (*P* < 0.05), as shown in Table 1.

After operation, in open field test, compared with control group, the number of standing times decreased, and the time staying in the central square increased in POCD group (*P* < 0.05), as shown in Table 2. However, compared with POCD group, the number of standing times increased, and the time staying in the central square decreased in POCD + Dex group (*P* < 0.05), as shown in Table 2.

### 3.2. The Number of A*β*, PSD95, and p-Tau Protein Positive Neurons in Hippocampus and Prefrontal Cortex from Aged Rats

**Figure 3:**
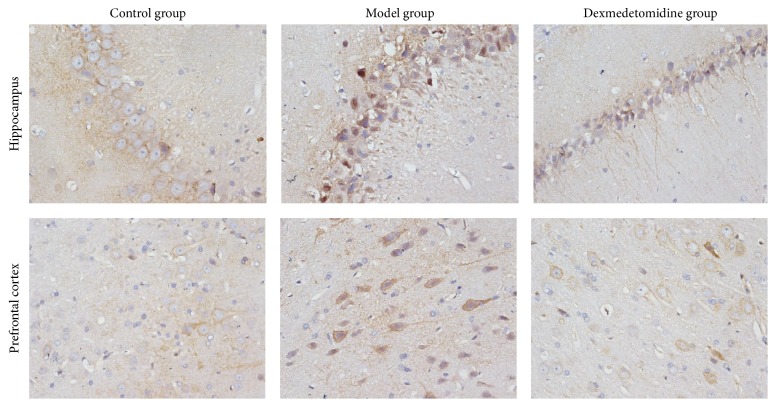
The immune-histochemical photos of A*β* positive cells in rats brain tissues (×400).

**Figure 4:**
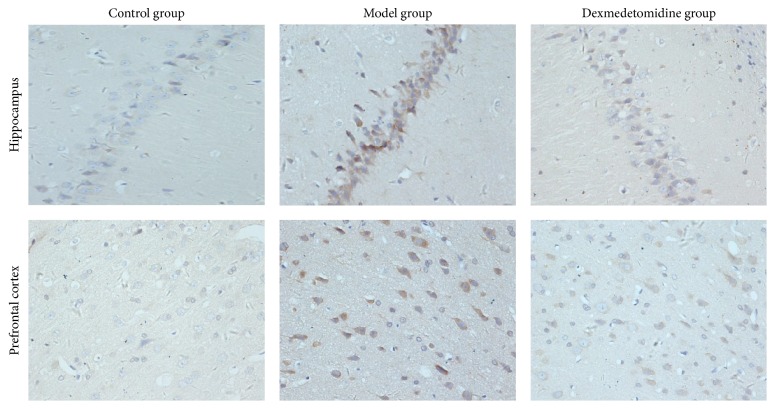
The immune-histochemical photos of p-Tau positive cells in rats brain tissues (×400).

**Figure 5:**
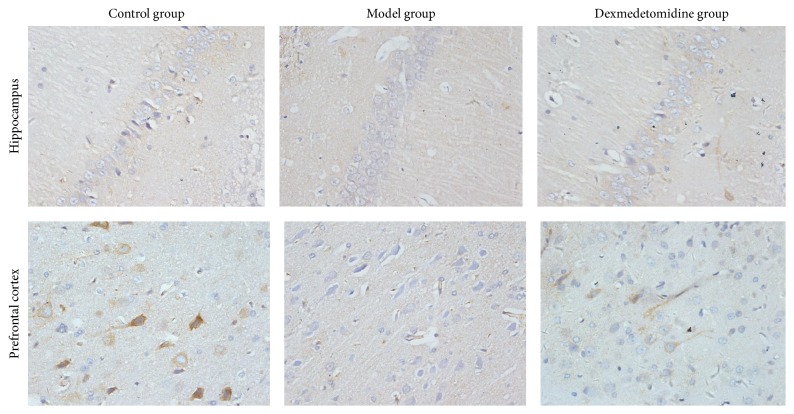
The immune-histochemical photos of PSD95 positive cells in rats brain tissues (×400).

After operation, in hippocampus and prefrontal cortex, compared with control group, the number of A*β* and p-Tau protein positive neurons increased, while the number of PSD95 positive neurons decreased in POCD group. However, compared with POCD group, the number of A*β* and p-Tau protein positive neurons decreased, while the number of PSD95 protein positive neurons increased in POCD + Dex group, as shown in Table 3, Figures 3–5.

### 3.3. The Expression of A*β* Protein, PSD95, and p-Tau Protein in Cerebrospinal Fluid from Aged Rats

After operation, compared with control group, the expression of p-Tau protein increased, while A*β* protein and PSD95 decreased in cerebrospinal fluid of POCD group (*P* < 0.05). However, compared with POCD group, the expression of p-Tau protein decreased, while A*β* protein and PSD95 increased in POCD + Dex group (*P* < 0.05), as shown in Table 4.

## 4. Discussion

For POCD patients, they have no mental disorder before surgery. However, after anesthesia and surgery, the impairments of memory, orientation, and mental concentration happen in these patients [13]. It is generally believed that cardiovascular surgery is a high risk for POCD. Some studies showed that, in hospital, the occurrence of cognitive decline was 53% for coronary artery bypass grafting (CABG) patients, 6 weeks after surgery was 36%, and 6 months after surgery was still 24% [14]. And the use of extracorporeal circulation operation during surgery is the main reason for the high risk of POCD [15]. In this study, the Sprague-Dawley rats, aged 18~20 months, underwent the extracorporeal circulation operation to mimic the clinical conditions of POCD. In Morris water-maze test, shuttle box test, and open field test, our results showed that, compared with control group, the escape latency, the latency of the initiative and passive avoiding, and the time staying in the central square increased, while the frequency of crossing the original platform, the times of initiative avoiding, and the number of standing times decreased in POCD group. Therefore, the learning, memory, adaptability, and cognitive abilities were impaired in these rats who undergone the extracorporeal circulation operation. And cognitive dysfunction phenotype had been produced successfully in POCD group. Meanwhile, the results also showed that, compared with POCD group, the escape latency, the latency of the initiative and passive avoiding, and the time staying in the central square decreased, while the frequency of crossing the original platform, the times of initiative avoiding, and the number of standing times increased in POCD + Dex group. Therefore, our study indicated that dexmedetomidine could improve the learning, memory, adaptability, and cognitive abilities after extracorporeal circulation operation in aged rats.

Some previous studies showed that the decrease of neuron number, cognitive related neurotransmitters (such as acetylcholine and glutamate), and corresponding receptors (such as NMDA receptor) might cause change of synaptic plasticity, thereby finally leading to cognitive function decline [16, 17]. In addition, some reports indicated that these abnormalities did not occur in one region of brain, but in the whole brain, including the prefrontal, parietal, cingulate, and hippocampus [18]. In the whole brain area, the hippocampus and prefrontal cortex were the most widely studied areas [19, 20]. Therefore, the hippocampus and prefrontal cortex were selected to investigate the mechanism of the effect of dexmedetomidine on POCD.

Some studies have reported that A*β* was produced by hydrolysis of amyloid precursor protein (APP). A*β* aggregates are shown to damage cell membrane integrity, increase intracellular calcium concentration, and induce apoptosis. And the excessive deposition of A*β* can induce neurotoxicity, which is positively correlated with the content in the brain [21]. Cytological studies and animal experiments showed that A*β* protein played a toxic role in neurons, which eventually led to degenerative changes in the nervous system [22]. A*β* protein can induce NMDA receptor-mediated intracellular Ca^2+^ elevation, thereby leading to neuronal cell damage and death [23, 24]. In addition, Tau protein exists in the vertebrate central and peripheral nervous system axons. Phosphorylation of Tau protein (p-Tau) is a decisive factor in A*β* protein mediated neuronal cell death. And p-Tau may lead to nerve cell death and degenerative change in nervous system [25, 26]. Finally, PSD95, which reflects synaptic activity and synaptic changes, can be combined with the NMDAR subunit of NR2 to form PSD95/NR2 complex by PDZ domain. The complex helps to maintain the normal function of NMDA receptor and regulate glutamate reuptake, neurotransmitters release, synapse formation, and transfer [27, 28]. In our study, we found that, compared with control group, the number of A*β* and p-Tau protein positive neurons increased, while the number of PSD95 positive neurons decreased in hippocampus and prefrontal cortex in POCD group. However, compared with POCD group, the number of A*β* and p-Tau protein positive neurons decreased, while the number of PSD95 positive neurons increased in POCD + Dex group. Therefore, our study indicated that dexmedetomidine could enhance the expression of PSD95 and reduce the expression of A*β* and p-Tau protein in hippocampus and prefrontal cortex and then to improve learning, memory, adaptability, and cognitive abilities after extracorporeal circulation operation in aged rats.

Finally, we detected the expression of A*β*, p-Tau, and PSD95 protein in the CSF in aged rats. Our results showed that A*β* and PSD95 decreased, while p-Tau protein increased in CSF from POCD group compared with control group. A*β* and p-Tau protein were regarded as Alzheimer's disease (AD) biomarkers, and POCD was identified after coronary artery bypass surgery with declined A*β* and increased p-Tau in cerebrospinal fluid revealing a unifying pathognomonic factor between POCD and AD [29].

In addition, compared with POCD group, A*β* protein and PSD95 increased, while p-Tau protein decreased in CSF from POCD + Dex group. The expression of PSD95 and p-Tau protein was consistent with those in hippocampus and prefrontal cortex. However, we found that the expression of A*β* protein in CSF was opposed to those in hippocampus and prefrontal cortex. Other studies have also reported that the concentration of A*β* protein was different between CSF and cortical brain biopsy. The accumulated A*β* protein in brain was high, while A*β* protein in CSF was low in Alzheimer disease [30], which is in line with the data shown earlier by postmortem brain [31, 32]. The possible mechanism of this phenomenon still remains unclear, and we propose that A*β* protein in the cerebrospinal fluid is accumulated in the corresponding brain region, thereby causing the reduction of A*β* protein in the cerebrospinal fluid.

In this study, we found that dexmedetomidine could affect the expression of A*β*, p-Tau, and PSD95 protein in CSF, hippocampus, and prefrontal cortex, which has been suggested to improve learning, memory, adaptability, and cognitive abilities after extracorporeal circulation operation in aged rats. Dexmedetomidine, as a highly selective *α* -2-adrenergic agonist, can function on neuronal presynaptic membrane receptor in locus coeruleus nucleus. It can inhibit the release of norepinephrine and the excessive excitement of neuronal synapses caused by operation. And we proposed that dexmedetomidine can inhibit the inflammatory response to protect the material transfer function of neurons axon and reduce the metabolic disorders of A*β*42 protein and Tau protein, even A*β*42 converted from APP (amyloid precursor protein) by *β*-secretase. This may explain the mechanism of the effect of dexmedetomidine and provide a way for preventing and/or treating POCD.

In conclusion, POCD in aged rats after extracorporeal circulation operation may result from upregulation of A*β* protein and p-Tau protein and downregulation of PSD95 in hippocampus and prefrontal cortex. The dexmedetomidine is proposed to be able to regulate expression of A*β*, p-Tau, and PSD95 in hippocampus and prefrontal cortex, which play a vital role in improving POCD. However, further investigations on how dexmedetomidine regulates expression of A*β*, p-Tau, and PSD95 protein in different brain regions are suggested to elucidate the detailed regulation mechanism.

## Figures and Tables

**Table 1:** The comparison of the shuttle box test in three groups.

Group	Times of avoiding (times)	Latency of the initiative avoiding (s)	Latency of the passive avoiding (s)
C	10.4 ± 2.7	4.7 ± 1.4	9.5 ± 1.7
M	4.9 ± 1.9^*∗*^	6.8 ± 2.9^*∗*^	11.9 ± 3.6^*∗*^
D	7.8 ± 2.5^#^	5.2 ± 1.9^#^	10.0 ± 2.0^#^

C: control group; M: POCD group; D: POCD + Dex group. ^*∗*^Compared with control group; ^#^compared with POCD group. *P* < 0.05 was considered as statistical significance.

**Table 2:** The comparison of the open field test in three groups.

Group	The number of standing times	The time staying in the central square (s)
C	10.3 ± 2.0	5.2 ± 0.8
M	6.4 ± 1.0^*∗*^	7.8 ± 1.5^*∗*^
D	9.5 ± 0.8^#^	6.1 ± 1.7^#^

C: control group; M: POCD group; D: POCD + dexmedetomidine group. ^*∗*^Compared with control group; ^#^compared with POCD group. *P* < 0.05 was considered as statistical significance.

**Table 3:** The comparison of the expressing of target protein in brain tissue in three groups.

group	Number of A*β* positive cells		Number of p-Tau positive cells		Number of PSD95 positive cells	
	hippoca	prefrontal	hippoca	prefrontal	hippocam	prefrontal
	mpus	cortex	mpus	cortex	pus	cortex
C	19 ± 13	38 ± 11	40 ± 11	48 ± 8	23 ± 5	37 ± 4
M	55 ± 13^*∗*^	59 ± 16^*∗*^	62 ± 20^*∗*^	76 ± 11^*∗*^	6 ± 3^*∗*^	16 ± 4^*∗*^
D	30 ± 9^#^	46 ± 12^#^	41 ± 10^#^	65 ± 11^#^	17 ± 7^#^	26 ± 2^#^

C: control group, M: POCD group, D: POCD + Dex group, ^*∗*^compared with control group, ^#^compared with POCD group, *P* < 0.05 was considered as statistical significance.

**Table 4:** The comparison of the content of target protein in cerebrospinal fluid in three groups.

Group	A*β* protein (pg/ml)	p-Tau protein (pg/ml)	PSD95 protein (pg/ml)
C	1272.8 ± 91.4	124.6 ± 11.2	62.4 ± 9.2
M	1152.0 ± 55.8^*∗*^	167.2 ± 30.3^*∗*^	42.1 ± 4.2^*∗*^
D	1211.6 ± 88.1^#^	132.8 ± 15.9^#^	57.1 ± 13.8^#^

C: control group; M: POCD group; D: POCD + Dex group. ^*∗*^Compared with control group; ^#^compared with POCD group. *P* < 0.05 was considered as statistical significance.
